# A Case of Facial Nerve Palsy As the Initial Presentation of Early Neurosyphilis: A Diagnostic Challenge

**DOI:** 10.7759/cureus.100936

**Published:** 2026-01-06

**Authors:** Tamara Kewan, Yaron River

**Affiliations:** 1 Neurology, Hillel Yaffe Medical Center, Hadera, ISR

**Keywords:** cranial neuropathy, facial nerve palsy, jarisch-herxheimer reaction, men who have sex with men, neurosyphilis, treponema pallidum

## Abstract

Neurosyphilis remains a diagnostic challenge, particularly when it presents with cranial neuropathies that mimic more common conditions such as Bell’s palsy, underscoring the need for heightened clinical awareness amid the global resurgence of syphilis, especially among men who have sex with men (MSM). We report the case of a 40-year-old, previously healthy man who presented with acute-onset left peripheral facial nerve palsy, initially treated as Bell’s palsy with corticosteroids; however, subtle neurological findings and limited improvement prompted further evaluation. Serologic testing revealed a new diagnosis of syphilis, cerebrospinal fluid analysis confirmed neurosyphilis, and brain MRI demonstrated characteristic facial nerve enhancement. The patient showed rapid clinical improvement following treatment with intravenous penicillin G, though his course was complicated by a Jarisch-Herxheimer reaction. This case emphasizes the importance of considering neurosyphilis in the differential diagnosis of cranial neuropathies, particularly in at-risk populations, as early recognition and appropriate antimicrobial therapy are vital for optimal neurological outcomes.

## Introduction

Neurosyphilis, caused by Treponema pallidum invasion of the CNS, can occur at any stage of syphilis infection and presents with diverse neurological manifestations [1]. While the advent of penicillin dramatically reduced the incidence of neurosyphilis in the mid-20th century, recent decades have witnessed a resurgence of syphilis globally, with corresponding increases in neurosyphilis cases [2, 3]. This resurgence has been particularly pronounced among men who have sex with men (MSM) and individuals with HIV infection [1, 2]. Cranial nerve (CN) involvement is a well-recognized but often underappreciated manifestation of neurosyphilis. Facial nerve (CN VII) palsy can be the initial or sole presenting feature, potentially leading to misdiagnosis as idiopathic Bell’s palsy or other more common etiologies [4, 5, 6]. The vestibulocochlear nerve (CN VIII) is also frequently affected, sometimes in combination with other CNs [7, 8]. Delayed diagnosis may result in permanent neurological sequelae, emphasizing the importance of early recognition and treatment [9, 10].

This case report describes a patient with early neurosyphilis presenting primarily with unilateral facial nerve palsy, initially treated as Bell’s palsy, whose additional neurological signs prompted comprehensive investigation leading to the correct diagnosis. The report highlights the diagnostic approach, neuroimaging findings, and treatment course, including the Jarisch-Herxheimer reaction, and reviews relevant literature to guide clinicians in recognizing this treatable condition.

## Case presentation

A 40-year-old divorced man with no significant past medical history presented to the emergency department with sudden-onset left-sided facial weakness that began the day prior. He reported difficulty gargling water and noticed facial asymmetry in the mirror, accompanied by left-sided facial pain, dizziness, neck tension, and recent episodes of left-sided otalgia and numbness. Two weeks earlier, he had experienced a brief flu-like illness. He denied limb weakness, gait instability, visual changes, hearing loss, rashes, or any history of recent insect or animal bites, foreign travel, or known tick exposure. His social history was notable for being a man who has sex with men with multiple recent partners, and a routine syphilis screen performed six months earlier had been negative. He denied prior genital lesions, rashes, or other symptoms suggestive of primary or secondary syphilis, and his HIV status was not initially documented.

On examination, his vital signs and general physical assessment were normal, with no rashes, lymphadenopathy, or genital lesions. Neurological evaluation revealed a left-sided peripheral facial nerve palsy involving the frontalis, orbicularis oculi, and buccinator muscles, resulting in incomplete eye closure and inability to raise the left eyebrow. Additional findings included a diminished bilateral gag reflex with rightward uvular deviation, suggesting left-sided vagal nerve involvement. Subtle motor deficits were observed, including a right upper extremity pronator drift and mild right lower extremity weakness; however, the patient attributed these findings to chronic orthopedic pain, and they were not consistently reproducible on repeated examination. Sensory examination, gait, and coordination were intact, and reflexes were normal and symmetric.

Because the initial presentation was dominated by a unilateral lower motor neuron facial palsy, the working diagnosis at the time was idiopathic peripheral facial nerve palsy, or Bell’s palsy. An otolaryngology consultation was obtained, and the patient was started on prednisone at a dose of 1 mg/kg daily with a planned taper. This diagnosis was considered provisional, and while he was treated accordingly, further investigations were pursued to exclude alternative etiologies. Over the following 48 hours, clinical improvement was limited. The presence of additional neurological features, including involvement of another cranial nerve consistent with left-sided vagal nerve dysfunction and inconsistent contralateral motor findings, prompted expansion of the differential diagnosis to include demyelinating processes such as multiple sclerosis and myelin oligodendrocyte glycoprotein antibody-associated disease, infectious etiologies including Lyme disease, brucellosis, HIV, and syphilis, autoimmune or inflammatory conditions such as neuro-Behçet’s disease and neurosarcoidosis, and vascular causes including brainstem stroke and vasculitis. A comprehensive infectious workup was therefore initiated, including serologic testing for syphilis, Lyme disease, HIV, and Brucella.

**Figure 1:**
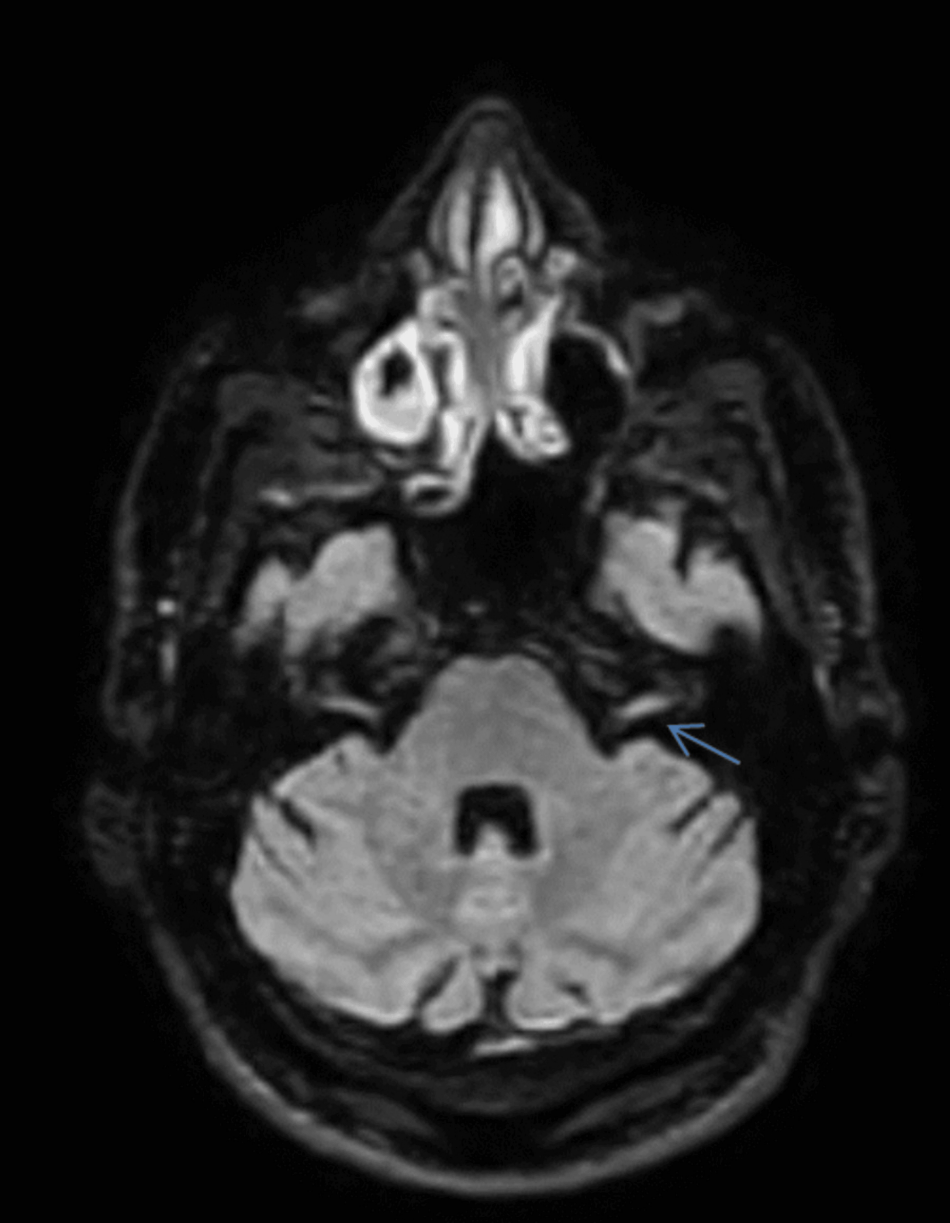
Axial T2-weighted FLAIR MRI of the brain demonstrating hyperintensity involving the left facial nerve (cranial nerve VII) in the cerebellopontine angle region (blue arrow). The increased signal intensity along the left facial nerve pathway is clearly visible compared with the normal-appearing right facial nerve. FLAIR: Fluid-Attenuated Inversion Recovery.

Serum studies returned positive for both non-treponemal and treponemal syphilis tests, while Lyme disease, HIV, and Brucella serologies were negative. Given the positive syphilis testing and neurological involvement, a lumbar puncture was performed. Cerebrospinal fluid was clear and colorless, with a white blood cell count of 4 cells/μL consisting entirely of lymphocytes, protein of 31.9 mg/dL, and glucose of 67.5 mg/dL, yielding a normal CSF-to-serum glucose ratio. CSF treponemal tests, including Enzyme-Linked Immunosorbent Assay (ELISA) and *Treponema pallidum* Hemagglutination Assay (TPHA), were positive, while the Venereal Disease Research Laboratory test (VDRL) was negative. In the context of neurological manifestations and positive serum syphilis serology, the positive CSF treponemal assays were diagnostic for neurosyphilis despite the negative VDRL, which is known to lack sensitivity in this condition. Brain MRI with and without contrast supported these findings by demonstrating characteristic Fluid-Attenuated Inversion Recovery (FLAIR) hyperintensity involving the left facial nerve (CN VII) (Figure 1).

Diffusion-weighted imaging revealed no restricted diffusion to suggest acute ischemia, effectively ruling out stroke as a contributing factor to the patient’s presentation. There was no abnormal leptomeningeal enhancement, no evidence of brainstem lesions, and no other parenchymal abnormalities, and the internal auditory canals appeared normal bilaterally. The radiological impression of unilateral facial nerve hyperintensity and enhancement was consistent with facial neuritis, supporting the diagnosis of neurosyphilis with cranial nerve involvement.

Based on the confirmed diagnosis of early neurosyphilis, the patient was admitted for intravenous antimicrobial therapy and initiated on a continuous infusion of aqueous crystalline penicillin G at 24 million units per day for 14 days, in accordance with established neurosyphilis treatment guidelines. Within several hours of the first penicillin dose, he developed an acute febrile reaction characterized by high fever, rigors, chills, profuse diaphoresis, a transient maculopapular truncal rash, and mild worsening of headache, features consistent with a Jarisch-Herxheimer reaction. This reaction resolved with supportive care and did not necessitate discontinuation of antibiotic therapy or the use of corticosteroids.

The patient completed the full 14-day course of penicillin without further complications, and serial neurological examinations demonstrated progressively improving facial nerve function throughout his hospitalization. By the time of discharge, he exhibited marked recovery with near-complete resolution of symptoms.

## Discussion

Syphilis, caused by *Treponema pallidum*, has experienced a dramatic resurgence over the past two decades, particularly in high-income countries, with the CDC reporting a 74% increase in reported syphilis cases between 2015 and 2019 and persistently high rates through 2023 [1, 2]. This rise has been most pronounced among MSM, who account for the majority of newly reported infections in many regions [2, 3]. Neurosyphilis can occur at any stage of syphilis infection and, although traditionally associated with late-stage disease, early neurosyphilis occurring within the first year of infection is increasingly recognized, especially in individuals with HIV [2, 11]. Determining the true incidence of neurosyphilis is challenging because many cases are asymptomatic or undiagnosed, but recent studies suggest that 5-10% of untreated syphilis cases may ultimately involve the nervous system [1, 12]. Coinfection with HIV is common, with 20-70% of neurosyphilis cases occurring in HIV-positive individuals, and HIV may accelerate syphilis progression; however, neurosyphilis also occurs frequently in HIV-negative individuals, as demonstrated in this case [2, 11].

Cranial nerve involvement is a well-documented but often under-recognized manifestation of neurosyphilis [4-6]. The facial (CN VII) and vestibulocochlear (CN VIII) nerves are most commonly affected, likely due to their anatomic proximity to the meninges and course through the temporal bone [7, 8, 13]. Numerous case reports describe facial nerve palsy as the initial or sole presenting sign of neurosyphilis, including presentations mimicking acoustic neuroma, Ramsay Hunt syndrome, or idiopathic Bell’s palsy [4-7, 14]. Bösel J et al. (2006) described a case of HIV-associated neurosyphilis mimicking acoustic neuroma, with facial and vestibulocochlear nerve involvement [7]. Maeda T et al. (2015) reported a case initially misdiagnosed as Ramsay Hunt syndrome (herpes zoster oticus) that was ultimately found to be neurosyphilis [4]. Ting CH et al. (2015) described bilateral facial nerve palsy as the sole initial symptom of syphilis in a patient who was subsequently diagnosed with neurosyphilis [5].

Proposed mechanisms for cranial nerve involvement include direct spirochetal invasion, meningeal inflammation at the skull base, vasculitis of the vasa nervorum, producing ischemic neuropathy, and, more rarely, gummatous compression lesions [7, 8, 15]. In the present case, MRI findings of facial nerve enhancement without significant meningeal involvement suggested a combination of direct neural inflammation and early meningovascular changes.

The clinical presentation of neurosyphilis is notoriously variable, contributing to its reputation as “the great imitator” [1, 12]. Early neurosyphilis may manifest with meningitis, meningovascular disease, ocular involvement, or auditory and vestibular symptoms [1, 8, 12]. Our patient presented with an acute unilateral facial palsy that closely resembled idiopathic Bell’s palsy, the most common cause of facial paralysis [16]. However, several red flags, including multi-cranial nerve involvement, contralateral motor findings, incomplete response to corticosteroids, high-risk sexual behavior, and a recent flu-like illness, suggested an alternative diagnosis. The differential diagnosis for acute facial palsy is broad and includes infectious causes such as Bell’s palsy, Ramsay Hunt syndrome, Lyme disease, neurosyphilis, HIV-associated neuropathy, otitis media, and tuberculosis; autoimmune conditions such as Guillain-Barré syndrome or sarcoidosis; neoplastic processes including schwannomas or meningeal carcinomatosis; vascular etiologies such as brainstem stroke; and traumatic or iatrogenic causes. Distinguishing features of neurosyphilis-related facial palsy include frequent bilateral involvement, association with other cranial neuropathies, auditory symptoms, positive serologic and CSF findings, MRI evidence of cranial nerve or meningeal enhancement, and occurrence in at-risk populations (MSM, HIV-positive, sex workers) [2-5, 8, 13, 14].

Diagnosing neurosyphilis relies on clinical suspicion supported by serum testing, CSF analysis, and neuroimaging [1, 10, 12]. Serologic evaluation requires both non-treponemal tests such as Rapid Plasma Reagin (RPR) or VDRL and treponemal tests such as *Treponema pallidum* Particle Agglutination (TP-PA) or Fluorescent Treponemal Antibody Absorption (FTA-ABS). While a positive treponemal test confirms syphilis exposure and a positive non-treponemal test suggests active infection, these results alone cannot distinguish systemic infection from neurosyphilis, making CSF evaluation essential in symptomatic patients. In some cases where lumbar puncture is contraindicated or refused, empiric neurosyphilis treatment may be justified given the significant risk of undertreatment. CSF studies typically include cell count, protein, glucose, VDRL, and treponemal assays [1, 10, 12]. Classic findings include lymphocytic pleocytosis, elevated protein, normal glucose, a positive CSF VDRL, and positive treponemal tests. However, the CSF VDRL, while highly specific, is only 30-70% sensitive, meaning neurosyphilis cannot be excluded based on a negative result [10, 12]. In this patient, the negative CSF VDRL contrasted with positive CSF treponemal tests, positive serum syphilis serology, cranial neuropathies, supportive MRI findings, and high-risk exposures, all of which together strongly supported the diagnosis. Some experts recommend calculating a CSF *Treponema pallidum* Hemagglutination Assay (TPHA) index or CSF/serum antibody ratio to confirm intrathecal antibody production, which may increase diagnostic specificity [7, 10].

Neuroimaging is not required for diagnosis but can provide valuable supportive information and help exclude alternative causes [8, 13, 15]. MRI may reveal meningeal enhancement, cranial nerve enhancement, vascular inflammation, gummatous lesions, or white matter abnormalities. In this case, T2/FLAIR hyperintensity and enhancement of the left facial nerve were consistent with cranial nerve inflammation and aligned with findings from reported cases of neurosyphilis-related facial and vestibulocochlear neuropathies [7, 8, 13]. Smith MM and Anderson JC (2000) described the MRI features of neurosyphilis affecting the facial and vestibulocochlear nerves, noting that enhancement patterns correlated with clinical deficits and often resolved after treatment [8]. Chandrasekharan R et al. (2022) reported similar findings in otosyphilis, emphasizing the value of MRI in localizing cranial nerve involvement and excluding alternative diagnoses such as schwannomas or meningiomas [13].

Treatment for neurosyphilis requires intravenous aqueous crystalline penicillin G at 18-24 million units per day for 10-14 days, administered every four hours or as a continuous infusion, as recommended by CDC and other guideline bodies [1, 10]. Alternative therapies include procaine penicillin with probenecid or ceftriaxone in patients unable to receive penicillin, though data supporting these alternatives are more limited. Post-treatment follow-up involves serial clinical evaluations and repeat non-treponemal serology at 3, 6, 12, and 24 months, with an expected fourfold decline in titers within 6-12 months. Repeat lumbar puncture is advised at six months if initial CSF abnormalities were present, clinical response is suboptimal, or serologic titers fail to improve appropriately [1, 10, 12].

The Jarisch-Herxheimer reaction is a well-known inflammatory response that can occur within hours of starting antimicrobial therapy for spirochetal infections. Characterized by fever, chills, headache, myalgias, hypotension, and transient worsening of symptoms, the reaction has been reported in approximately 30-40% of neurosyphilis cases and is mediated by cytokine release following spirochetal lysis [4, 17]. Management is supportive, and antibiotic therapy should not be interrupted. In this patient, a Jarisch-Herxheimer reaction emerged within hours of the first penicillin dose but resolved quickly with symptomatic care, supporting the diagnosis of active treponemal infection.

The prognosis of neurosyphilis varies depending on the timeliness of diagnosis, the extent of neurological involvement, HIV status, and the duration of symptoms before treatment [9, 10]. Cranial neuropathies often respond well to therapy, with multiple reports documenting rapid improvement of facial nerve function after penicillin treatment [4-7, 9]. Auditory symptoms, however, may recover incompletely. Our patient experienced near-complete resolution of facial palsy by the end of the 14-day treatment course, consistent with early neurosyphilis managed promptly.

This case also illustrates several important public health implications. Regular screening of at-risk populations, especially sexually active MSM, partner notification and treatment, and universal HIV testing in patients diagnosed with syphilis are essential components of disease control [1, 2]. Preventive counseling on safe sexual practices and appropriate STI screening intervals is crucial, as is heightened clinical awareness of syphilis and neurosyphilis in patients presenting with neurological symptoms in high-risk groups [1, 3].

## Conclusions

This case demonstrates how neurosyphilis can initially present as peripheral facial nerve palsy and closely mimic idiopathic Bell’s palsy, delaying appropriate diagnosis. The combination of atypical neurological findings, high-risk exposures, positive serology, supportive CSF studies, and characteristic MRI enhancement ultimately clarified the underlying etiology and guided effective treatment. Early recognition in this patient allowed timely initiation of intravenous penicillin, resulting in substantial neurological recovery. Notably, this peripheral facial nerve palsy represented this patient’s first clinical manifestation of syphilis, occurring despite negative syphilis testing performed six months prior to presentation.

The clinical course further underscores the importance of considering neurosyphilis when cranial neuropathies present with additional neurological signs or fail to respond to standard therapy. Careful integration of clinical, laboratory, and imaging findings enabled accurate diagnosis and successful management, highlighting the value of thorough evaluation in preventing avoidable long-term deficits.

## References

[1] Wu S, Ye F, Wang Y, Li D (2024). Neurosyphilis: insights into its pathogenesis, susceptibility, diagnosis, treatment, and prevention. Front Neurol.

[2] Ha T, Tadi P, Leslie SW, Dubensky L (2025). Neurosyphilis. https://www.ncbi.nlm.nih.gov/books/NBK540979/.

[3] Centers for Disease Control and Prevention (2023). Sexually Transmitted Infections Surveillance 2022. Sexually Transmitted Disease Surveillance.

[4] Maeda T, Yoshizawa S, Hirayama T, Saga T, Tateda K, Urita Y (2015). Neurosyphilis mimicking Ramsay Hunt syndrome. J Nippon Med Sch.

[5] Ting CH, Wang CW, Lee JT, Peng GS, Yang FC (2015). Bilateral facial nerve palsy as the sole initial symptom of syphilis: a case report. CJEM.

[6] Njiru E, Abdulkadir J, Kamuren Z, Kigen G (2017). Early neurosyphilis presenting with facial palsy and an oral ulcer in a patient who is human immunodeficiency virus positive: a case report. J Med Case Rep.

[7] Bösel J, Klingebiel R, Schielke E (2006). HIV-associated neurosyphilis mimicking acoustic neurinoma. J Neurol.

[8] Smith MM, Anderson JC (2000). Neurosyphilis as a cause of facial and vestibulocochlear nerve dysfunction: MR imaging features. AJNR Am J Neuroradiol.

[9] Abkur TM, Ahmed GS, Alfaki NO, O'Connor M (2015). Neurosyphilis presenting with a stroke-like syndrome. BMJ Case Rep.

[10] (2024). Syphilis 2024: Updated Guideline. https://www.bashh.org/resources/25/updated_guideline_syphilis_2024/.

[11] Ghanem KG, Moore RD, Rompalo AM, Erbelding EJ, Zenilman JM, Gebo KA (2008). Neurosyphilis in a clinical cohort of HIV-1-infected patients. AIDS.

[12] Ropper AH (2019). Neurosyphilis. N Engl J Med.

[13] Chandrasekharan R, Kulkarni C, Pullara SK, Moorthy S (2022). Magnetic resonance imaging in otosyphilis: a rare manifestation of neurosyphilis. Indian J Radiol Imaging.

[14] Alqahtani S (2014). Acute cranial neuropathies heralding neurosyphilis in human immunodeficiency virus-infected patient. Am J Case Rep.

[15] 15] Brightbill TC, Ihmeidan IH, Post MJ (1995). Neurosyphilis in HIV-positive and HIV-negative patients: neuroimaging findings. AJNR Am J Neuroradiol.

[16] Baugh RF, Basura GJ, Ishii LE (2013). Clinical practice guideline: Bell's palsy. Otolaryngol Head Neck Surg.

[17] Butler T (2017). The Jarisch-Herxheimer reaction after antibiotic treatment of spirochetal infections: a review of recent cases and our understanding of pathogenesis. Am J Trop Med Hyg.

